# Gastroprotective Effects of Lion's Mane Mushroom *Hericium erinaceus* (Bull.:Fr.) Pers. (Aphyllophoromycetideae) Extract against Ethanol-Induced Ulcer in Rats

**DOI:** 10.1155/2013/492976

**Published:** 2013-11-05

**Authors:** Jing-Yang Wong, Mahmood Ameen Abdulla, Jegadeesh Raman, Chia-Wei Phan, Umah Rani Kuppusamy, Shahram Golbabapour, Vikineswary Sabaratnam

**Affiliations:** ^1^Mushroom Research Centre, University of Malaya, 50603 Kuala Lumpur, Malaysia; ^2^Institute of Biological Sciences, Faculty of Science, University of Malaya, 50603 Kuala Lumpur, Malaysia; ^3^Department of Biomedical Science, Faculty of Medicine, University of Malaya, 50603 Kuala Lumpur, Malaysia

## Abstract

*Hericium erinaceus* is a famous tonic in oriental medicine. The gastroprotective effects of aqueous extract of *H. erinaceus* against ethanol-induced ulcers in *Sprague Dawley* rats were investigated. The possible involvements of lipid peroxidation, superoxide dismutase, and catalase were also investigated. Acute toxicity study was performed. The effects of aqueous extract of *H. erinaceus* on the ulcer areas, ulcer inhibition, gastric wall mucus, gross and histological gastric lesions, antioxidant levels, and malondialdehyde (MDA) contents were evaluated in ethanol-induced ulcer *in vivo*. In acute toxicity study, a high dose of 5 g/kg did not manifest any toxicological signs in rats. The extract promoted ulcer protection as ascertained by a significant reduction of the ulcer area. Furthermore, it exhibited a significant protection activity against gastric mucosal injury by preventing the depletion of antioxidant enzymes. The level of MDA was also limited in rat stomach tissues when compared with the ulcer control group. Immunohistochemistry showed upregulation of HSP70 protein and downregulation of BAX protein in rats pretreated with the extract. The aqueous extract of *H. erinaceus* protected gastric mucosa in our *in vivo* model. It is speculated that the bioactive compounds present in the extract may play a major role in gastroprotective activity.

## 1. Introduction

Peptic ulcer, including both gastric and duodenal ulcers, has represented a major threat to the world's population over the past two centuries. Chronic inflammation due to colonisation of *Helicobacter pylori* and nonsteroidal anti-inflammatory drugs account for the large majority of peptic ulcer disease [1]. In 2007, around 14.5 million persons in the United States suffered from peptic ulcers, and this number was increased to 14.99 million in 2010 [2, 3]. Gastric ulcer is a serious gastrointestinal disorder. It occurs when the gastric mucosa is impaired, leading to perforations of the stomach lining followed by stomach wall bleeding. Generally, ulcers result from an imbalance between increased aggressive factors, such as acid and pepsin secretions [4], and decreased defensive factors, such as mucus and bicarbonate secretions [5], mucosal barrier [6], mucosal blood flow [7], and endogenous prostaglandin production [8]. Treatment strategies that address one or more of these factors include amelioration of pain, limitation of complications, healing promotion, and prevention of recurrence. Current treatment strategy is mainly aimed to suppress the secretion of gastric acids, which is considered as the main cause of ulcer formation [1]. 

As patients experience unpleasant side effects of the long-term use of commercially available drugs, the search for natural therapeutic agent to prevent gastric ulceration is still ongoing. Natural products from herbs, medicinal plants, spices, vegetables, and crude drug substances are considered to be potential sources to fight various diseases including gastric ulcers. Some of the medicinal plants such as turmeric (*Curcuma longa*) [9], *Flabellaria paniculata* [10], *Cassia sieberiana* roots bark [11], *Mucuna pruriens* leaf [12], *Phyllanthus niruri* leaf [13], *Polygonum chinense* leaf [14], *Ocimum suave* leaf [15], ginger (*Zingiber officinale* Roscoe) [16], and *Hericium erinaceus* fruiting body [17] were reported to have antiulcer activity. 

Many mushrooms have a long history of applications in traditional oriental therapies. Modern clinical practice in countries such as Japan, China, and Korea continues to rely on mushroom-derived preparations. Mushrooms reported to possess anti-ulcer properties include *Lentinula edodes* (Berk.) Pegler, *Pleurotus ostreatus* (Jacq.) P. Kumm [18], and *Ganoderma lucidum* (Fr.) P. Karst [19]. *H. erinaceus* (Bull.:Fr.) Pers. (Aphyllophoromycetideae) is a temperate mushroom that has been domesticated and is commercially grown in Malaysia. The cultures of *Hericium* spp. or their extracts processed in tablets are used for curing gastric ulcer and chronic gastritis [20, 21]. Our previous study demonstrated that this mushroom exhibited cytoprotection activity against ethanol-induced gastric ulcers in rats [17]. However, the mechanisms of gastric ulcer prevention and healing by *H. erinaceus* have not been reported. Thus, the present work aims to investigate the mechanisms of the gastroprotective effects of aqueous extract of *H. erinaceus* basidiocarp. The antioxidant parameters, that is, superoxide dismutase (SOD) and catalase (CAT), in the gastric tissue of rats were also studied. Further, the level of gastric mucosal malondialdehyde (MDA), which is thought to reflect free radical mediated cell membrane damage, was also accessed in the gastric tissues.

## 2. Materials and Methods

### 2.1. Chemicals

Assay kits for the activity of SOD and CAT were obtained from Cayman Chemical Company (MI, USA). All other chemicals used in this study were of analytical grade. Omeprazole was obtained from the University of Malaya Medical Centre (UMMC) Pharmacy.

### 2.2. Preparation of Mushroom Extract

Fresh basidiocarps (fruiting bodies) of *H. erinaceus* were obtained from Highland Mushroom Farm, Genting Highlands, Pahang, Malaysia. The basidiocarps were freeze-dried and subjected to aqueous extraction, according to Wong et al. [22].

### 2.3. Experimental Animals

Healthy male and female *Sprague Dawley* rats aged 6 to 8 weeks and weighed between 180 g and 200 g were used in the study. The female animals used were nulliparous and nonpregnant. Food and water were provided *ad libitum*. The animals were kept at 27°C (±2°C) and a 12 h light/dark cycle was maintained throughout the experimental period. Experimental protocols were approved by the ethical committee with Ethics number ISB/11/08/2011/WJY(R) under Laboratory Animal Science Centre, Faculty of Medicine, University of Malaya. All efforts were made to minimise both the number of animals used and unwanted stress or discomfort to the animals throughout the experiment. 

### 2.4. Acute Toxicity Studies

For each gender, 18 rats were randomly divided into three groups (*n* = 6), namely, normal control, low-dose, and high-dose groups. Normal control rats were administered orally with distilled water only, whereas low dose and high dose rats were fed orally with aqueous extract of *H. erinaceus* at dose levels of 2 g/kg and 5 g/kg body weight equivalent to a volume of 5 mL/kg body weight, according to OECD [23]. The rats were observed for 24 h and then for the next 14 days. During the experimental period, the rats were observed for signs of toxicity, morphological behavior, and mortality. 

### 2.5. Gastroprotective Activity

The gastroprotective activity of aqueous extract of *H. erinaceus* was performed in ethanol-induced ulcer model rats as described by Garg et al. [24]. The rats were divided into seven groups with six animals per group. Group 1 and 2 received distilled water and served as negative-control group and ulcer-control group, respectively. Group 3 received omeprazole (20 mg/kg) orally and served as the reference drug group for comparison. Animals in groups 4, 5, 6, and 7 received the extracts at the doses of 50, 100, 200, and 400 mg/kg bodyweight, respectively. After 1 h, all of the groups, except group 1, received 20 mL/kg of ethanol 95%. The animals were sacrificed 1 h later under anesthetised diethyl ether and their stomachs were quickly removed for further studies.

### 2.6. Gross Gastric Lesions Evaluation

Stomach of each experimental animal was opened along the greater curvature and rinsed with distilled water to remove gastric contents. Grossly, gastric ulcers appeared as elongated bands on the gastric mucosa with hemorrhagic lesions being parallel to the long axis of the stomach. The mucosa was assessed for damage under dissecting microscope (1.8×) and a planimeter was used to measure the ulcers area (hemorrhagic lesions). The length and width of each lesion were measured and the sum of the area of all lesions for each stomach was expressed as the ulcer area (mm^2^). The ulcer area (UA) was calculated as described by Kauffman Jr. Grossman [25]. The inhibition percentage (*I*%) was calculated using the formula

(1) $$\begin{array}{c} I\%=\left[\left({\text{UA}}_{\text{control}}-{\text{UA}}_{\text{treated}}\right)÷{\text{UA}}_{\text{control}}\right]\times 100\%. \end{array}$$

### 2.7. Mucus Production of the Gastric Mucosa

The gastric mucosa of each rat was gently scraped using a glass slide and the mucus obtained was weighed using a precision electronic balance.

### 2.8. Histological Evaluation of Gastric Tissues

The gastric wall samples were fixed in 10% (v/v) buffered formalin and processed to produce paraffin wax tissue sections. The stomach damage was assessed by examining 5 *μ*m sections stained with hematoxylin and eosin. 

### 2.9. Immunohistochemical Evaluation of Gastric Tissues

Tissue sections (prepared as given above) were heated at 60°C for 25 min in an oven (Venticell, MMM, Einrichtungen, Germany) and then deparaffinized in xylene and rehydrated using graded alcohol. Antigen retrieval process was done in 10 mM sodium citrate buffer boiled in a microwave. Immunohistochemical staining was performed according to manufacturer's protocol (Dakocytomation, USA). Positive findings of the immunohistochemical staining should be seen as brown stains under a light microscope.

### 2.10. Determination of Gastric Wall Mucus

The glandular segments from stomach, which had been opened along their greater curvature, were weighed. The quantity of alcian blue recovered from the glandular tissue was then calculated.

### 2.11. Biochemical Changes in Gastric Tissues

The gastric tissues were homogenised in 8 : 1 (w/v) phosphate buffer saline. After centrifugation at 12,000 ×g for 15 min at 4°C, the supernatant was stored at −80°C prior to analysis. The homogenates were used for the estimation of MDA level and SOD and CAT activities.

#### 2.11.1. Measurement of Malondialdehyde Content

The level of lipid peroxides in the gastric tissue homogenate was determined through the measurement of total thiobarbituric acid-(TBA-) reactive substance (Cayman Chemical, USA) at 532 nm (Shimadzu, Japan). Each assay was carried out in triplicate. The results were expressed as *μ*M 1,1,3,3-tetraethoxypropane equiv/g of wet tissue.

#### 2.11.2. Measurement of Superoxide Dismutase Activity

The SOD activity was assayed using SOD kit (Cayman Chemical, USA) following the manufacturer's instruction. Diluted radical detector solution (200 *μ*L) and samples (10 *μ*L) were added to microplate wells. Reaction was initiated by adding 20 *μ*L of diluted xanthine oxidase to the mixture followed by shaking for a few seconds to mix. The plate was then incubated on a shaker for 20 minutes at room temperature and absorbance was read at 440 nm using an ELISA reader.

#### 2.11.3. Measurement of Catalase Activity

For CAT assay (Cayman Chemical, USA), the diluted assay buffer (100 *μ*L), methanol (30 *μ*L), and sample (20 *μ*L) were added to wells. Diluted hydrogen peroxide (20 *μ*L) was then added to the mixture to initiate the reactions. The plate was covered and incubated on a shaker incubator for 20 min at room temperature. Diluted potassium hydroxide (30 *μ*L) was then added to each well to terminate the reaction followed by 30 *μ*L of catalase purpald (Chromagen). The plates were incubated for 10 min at room temperature on a shaker. Catalase potassium periodate (10 *μ*L) was then added to each well and reincubated for 5 min at room temperature with agitation. The plate was read at 540 nm using an ELISA reader.

### 2.12. Statistical Analysis

All values reported are mean ± SEM (*n* = 6). Statistical analysis was done using SPSS version 17.0. The significant differences amongst groups were assessed using one-way ANOVA and Tukey's Multiple Comparison Test. A value of *P* < 0.05 was considered significant.

## 3. Results

### 3.1. Acute Toxicity Study of Gastric Organs

There was no mortality of rats at all dose levels tested. This result showed that aqueous extract of *H. erinaceus* was well tolerated by experimental rats and safe even at high dose level of 5 g/kg of body weight. Our results confirmed the previously published study on acute toxicity of the plant [26].

### 3.2. Gross Evaluation of the Gastric Organs

In ulcer prevention experiment, group 2 animals, the ulcer control animals, showed severe mucosal injury with ulcer area 894.00 ± 36.69 mm^2^ (Table 1). A significant decrease (*P* < 0.05) in the ulcer area was observed in group 3 animals which were pre-treated with omeprazole (the reference drug group) (285.90 ± 19.64 mm^2^). For the groups pre-treated with the mushroom extracts, the ulcer was significantly (*P* < 0.05) decreased in a dose-dependent manner when compared to group 2. The ulcer area was significantly (*P* < 0.05) reduced from 894.00 ± 36.69 mm^2^ (control rats) to 240.00 ± 17.31 mm^2^ in the rats pre-treated with 400 mg/kg of mushroom extracts. As shown in Table 1, the rats pre-treated with 400 mg/kg of mushroom extract exhibited the highest inhibition percentage of ulcer area formation which was 72.97%, followed by omeprazole (67.45%), mushroom extracts of 200 mg/kg (48.60%), 100 mg/kg (32.17%), and 50 mg/kg (18.14%). The high dose pretreatment at 400 mg/kg of mushroom extract prior to ethanol treatment was effective in ulcer prevention. There was no significant difference in efficacy of 400 mg/kg of mushroom extract as compared to the reference drug group in this study.

### 3.3. Gastric Mucus Production of the Gastric Organs

Mucus production by gastric mucosa increased gradually in the experimental animals pre-treated with mushroom extracts in a dose-dependent manner (Table 1). Group 7 rats which were administrated with 400 mg/kg of mushroom extract produced the highest amount of mucus (1.18 ± 0.04 g) which was 1.49-fold higher (*P* < 0.05) than that of ulcer control group.

### 3.4. Histopathology of the Gastric Tissues

**Figure 1:**
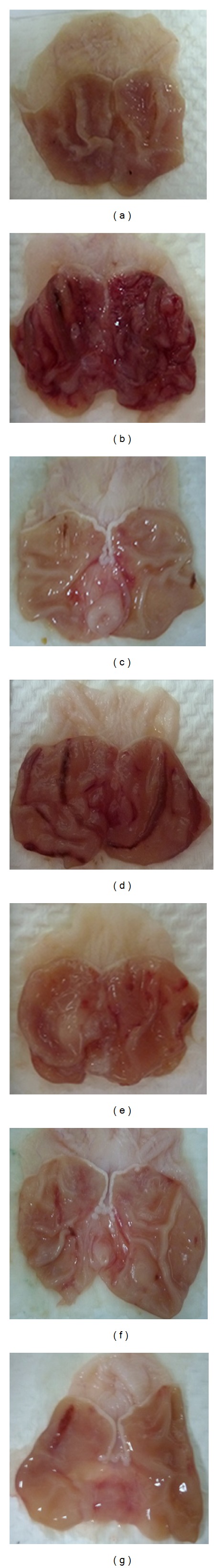
Protective effect of aqueous extract of *Hericium erinaceus* on gastric mucosal lesions induced by ethanol in rats. Macroscopic evaluation of the gastric mucosa in the rats pre-treated with 5 mL/kg distilled water (normal control) (a). Rats pretreated with 5 mL/kg distilled water (ulcer control) (b). Rats pre-treated with omeprazole (20 mg/kg, the reference drug group) (c). Rats pre-treated with the aqueous extract of *H. erinaceus* 50 mg/kg (d), 100 mg/kg (e), 200 mg/kg (f), and 400 mg/kg (g).

**Figure 2:**
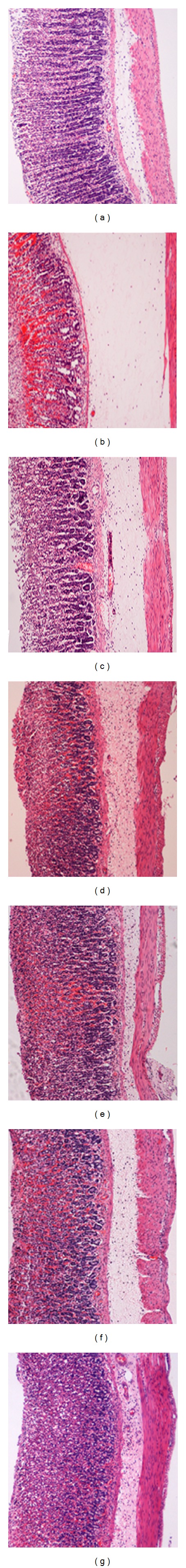
Effect of aqueous extract of *Hericium erinaceus* on histological evaluation in ethanol-induced ulcer model. Histology evolution of gross appearance of the gastric mucosa in rats pre-treated with 5 mL/kg distilled water (normal control) (a). Rats pre-treated with 5 mL/kg distilled water (ulcer control) (b). Rats pre-treated with omeprazole (20 mg/kg, the reference drug group) (c). Rats pre-treated with the aqueous extract of *H. erinaceus* 50 mg/kg (d), 100 mg/kg (e), 200 mg/kg (f), and 400 mg/kg (g).

The rats in the ethanol-induced ulcer control (group 2) showed extensive damage to the gastric mucosa, and necrotic lesions were found to penetrate deep into the mucosa. Extensive edema and leucocytes infiltration of the submucosal layer were also observed. Furthermore, the loss of normal glandular architecture of the stomach was observed (Figures 1 and 2). In animals treated with omeprazole (20 mg/kg), normal glandular pattern with mild inflammation and infiltration of leucocytes in the stomach was observed. The rats treated with mushroom extract at 200 mg/kg demonstrated comparatively better protection of the gastric mucosa as reduction or absence of ulcer area, and submucosal edema and leucocytes infiltration were observed. The rats pre-treated at 400 mg/kg showed near-normal architecture comparable to normal rats (Figures 1 and 2). 

### 3.5. Immunohistochemistry

**Figure 3:**
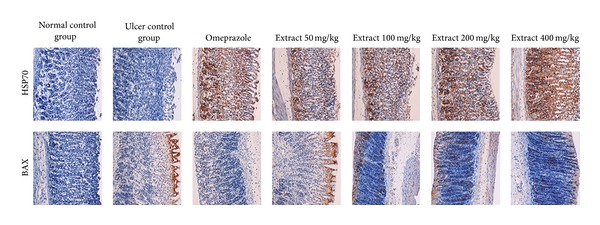
Immunohistochemical analysis of expression of HSP and BAX proteins in the stomach of rats in ethanol-induced gastric ulcer. Immunohistochemistry staining of HSP70 (first row) and BAX (second row) proteins showed overexpression of HSP70 protein and downexpression of BAX protein in rats pretreated with *H. erinaceus* extract (magnification 20×).

The rats pre-treated with aqueous extract of *H. erinaceus* in ethanol-induced injury rats expressed high level of heat shock protein (HSP) 70 (Figure 3). In contrast, the expression of HSP70 in ethanol-induced gastric tissues (group 2) was downregulated as compared to that of mushroom extract-treated groups (Figure 3). Immunohistochemical staining of BAX protein showed that the pretreatment with mushroom extract prior to ethanol-induced injury of rats caused downregulation of BAX protein expression. Furthermore, upregulation of BAX in ulcer control group was observed when compared to the mushroom-extract-treated groups. 

### 3.6. Gastric Wall Mucus

**Figure 4:**
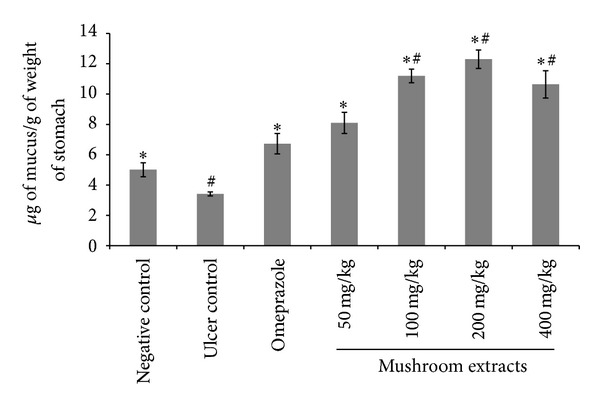
Effects of oral pretreatments of *H. erinaceus* extract on alcian blue binding to gastric mucous in rats. All values were expressed as mean ± standard error of mean. *Significant difference (*P* < 0.05) when compared with the ulcer control (group 2). ^#^Significant difference (*P* < 0.05) when compared with the omeprazole (group 3).

The aqueous extract of *H. erinaceus* significantly increased (*P* < 0.05) the alcian blue binding capacity of gastric wall mucus when compared to ulcer control group (Figure 4). Surprisingly, the pre-treatment with extract at 200 mg/kg showed the highest alcian blue binding capacity of gastric mucosa (12.29 ± 0.61 *μ*g of mucus/g weight of stomach), followed by 100 mg/kg extract group (11.19 ± 0.45 *μ*g of mucus/g weight of stomach). However, there was no significant difference between these two groups. 

### 3.7. Antioxidant Activity of Aqueous Extract of Hericium erinaceus

**Figure 5:**
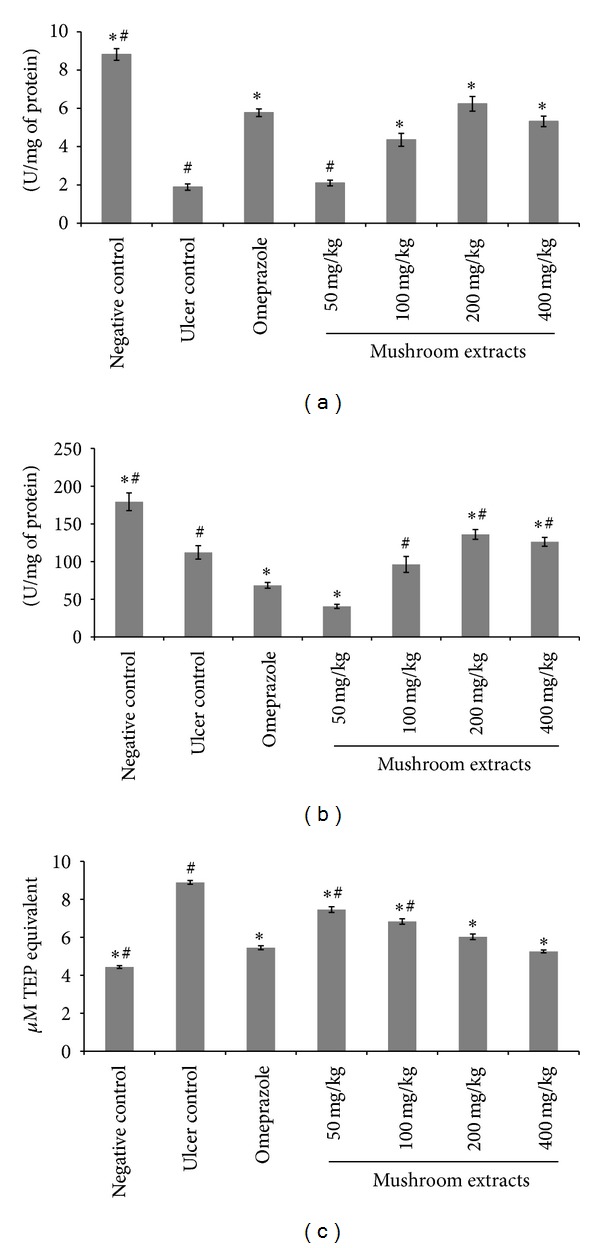
Effects of aqueous extract of* H. erinaceus* on the SOD (a) and CAT (b) activities and lipid peroxidation (c) in stomach of rats. All values were expressed as mean ± standard error of mean. *Significant difference (*P* < 0.05) when compared with the ulcer control (group 2). ^#^Significant difference (*P* < 0.05) when compared with the omeprazole (group 3).

As shown in Figure 5(a), SOD activity decreased significantly in the stomach tissue of the ulcer control group (1.89 ± 0.16 U/mg) as compared to the negative control (8.82 ±0.31 U/mg). A reduction of SOD activity indicated elevated production of reactive oxygen species (ROS) which caused gastric damage in ethanol-induced ulcer tissue. Omeprazole was found to prevent the decrease in SOD activity to 5.77 ± 0.2 U/mg. While the aqueous extract of *H. erinaceus* at 50 mg/kg did not significantly (*P* < 0.05) reduce SOD activity, it increased SOD activity significantly (*P* < 0.05) at the other doses (100, 200, and 400 mg/kg) tested compared to the control. On the other hand, mushroom extract was at 100, 200, and 400 mg/kg were also found to prevent the depletion of CAT activities (Figure 5(b)). The efficacy of mushroom extracts at 200 and 400 mg/kg was significantly higher (*P* < 0.05) than that of group 3. As stipulated in Figure 5(c), MDA production, which is an index of lipid peroxidation, was the highest (8.89 ± 0.89 *μ*M TEP equivalent) in group 2 due to severe oxidative stress induced by ethanol. Mushroom extracts at all doses tested decreased the MDA level in gastric tissue dose dependently and significantly (*P* < 0.05) as compared to the control. 

## 4. Discussion

The present study showed that pre-treatment with the aqueous extract of *H. erinaceus* protected the gastric mucosa and inhibited leucocytes infiltration of gastric wall in rats with ethanol-induced gastric ulcer. Cheng and Koo [27] and Hariprasath et al. [28] demonstrated that pre-treatment with plant extract before ethanol administration significantly decreased leucocytes and in particular neutrophils infiltration of gastric mucosa. Reduction of neutrophil infiltration into ulcerated gastric tissue was the indication of healing of gastric ulcers in rats [29]. Ethanol would damage the gastric mucosa and increase neutrophil infiltration into the gastric mucosa. Leucocytes, namely, the neutrophils, are a major source of inflammatory mediators and can release ROS such as superoxide-, hydrogen peroxide-, and myeloperoxidase-derived oxidants. These ROS are highly cytotoxic and can induce tissue damage [27]. Tsukimi et al. [30] found that suppression of neutrophil infiltration during inflammation could enhance gastric ulcer healing. Taken together, the aqueous extract of *H. erinaceus* may protect gastric mucosa viasuppression of neutrophil infiltration and antioxidant machinery.

HSPs play important roles in normal condition as well as pathological situations involving both systemic and cellular stress [31]. In the gastric mucosa, it has been reported that HSP70 has important cytoprotective functions *in vitro* and *in vivo* [32]. In accordance with several studies [33–35], the present finding showed remarkable upregulation of the expression of HSP70 in rats pre-treated with mushroom extracts. The HSP70 family functions as a molecular chaperone and reduces stress-induced denaturation and aggregation of intracellular proteins. ROS that are generated by ethanol suppress the expression of HSP70 and increase the expression of BAX protein, whereas HSP70 proteins protect cells from oxidative stress or heat shock. The expression of HSP70 observed in this study indicated that the aqueous extract of *H. erinaceus* protected the gastric tissues through the upregulation of HSP70.

Gastric mucus barrier plays an important role in gastroprotection [5]. The pre-treatment with aqueous extract of *H. erinaceus* significantly increased the gastroprotective effect, with the free mucus being significantly (*P* < 0.05) increased when compared to the mucus barrier of ulcer control animals. The mucus was comprised of mucin-type glycoprotein, which was detected by a dye called alcian blue [36]. Alcian blue dye binds with negatively charged materials. The mucus gel adhering to the gastric mucosal surface protects the underlying epithelium against acid, pepsin, and necrotising agents such as ethanol and indomethacin [37]. Nevertheless, Allen and Flemström [5] demonstrated that gastric wall mucus plays more crucial role in the defence of the gastric mucosa against aggression of chemical or mechanical factors than the soluble mucus in the lumen of the stomach. Gastric wall mucus coating might be alleviated in the repair of the damaged gastric epithelium [38]. Hence, the increase of alcian blue binding capacity suggested that aqueous extract of *H. erinaceus* was able to trigger the gastric mucus barrier defence system. 

Arafa and Sayed-Ahmed [39] found that the production of oxygen free radicals played a crucial role in the development of ethanol-induced gastric lesions. Huh et al. [40] also revealed that free radical mechanisms contributed to ethanol-induced tissue injury. Superoxide produced by peroxidase in the stomach tissues might damage cell membranes and might cause ulcer while increasing MDA level [41]. Preventive antioxidants, such as SOD and CAT enzymes, are the first line of defence against ROS. SOD scavenges the superoxide radical O_2_^−^, one of the ROS responsible for lipid peroxidation [42]. This reaction leads to an increase in the generation of peroxyl radical H_2_O_2_^−^, which is also capable of producing more oxidative damage [43]. Catalase and other peroxidases further reduce H_2_O_2_^−^. Treatment with the aqueous extract of *H. erinaceus* significantly (*P* < 0.05) decreased lipid peroxidation and increased SOD and CAT activities, indicating its efficacy in preventing free radical-induced damage by quenching free radicals in the gastric tissue of the ethanol-induced rats to exhibit gastroprotective activity. It is evident from the results of this study that antioxidant compounds mainly the phenolic compounds present in the aqueous extract of *H. erinaceus*, could play synergetic effect in experimental animals to protect against gastric tissue damage. In support of this, our previous study [44] had shown that *H. erinaceus* had potent antioxidant activities. Aqueous extracts of *H. erinaceus* contain mainly polysaccharides, phenolic compounds, and hericenones (unpublished data). Nevertheless, further studies determining the bioactive components responsible for the mechanism of anti-ulcer activities of *H. erinaceus* extracts are warranted.

## 5. Conclusion

The aqueous extract of *H. erinaceus* protected gastric mucosa against ethanol-induced injury. The protection was shown to be dose dependent according to the reduction of ulcer areas in the gastric wall and the reduction or inhibition of edema and leucocytes infiltration of submucosal layers, and protection was significant at the maximum dosage at 400 mg/kg. The extract also significantly increased the SOD and CAT activities and decreased the level of lipid peroxidation (as indicated by MDA level) in the gastric tissue homogenates. Immunohistochemistry analysis showed upregulation of HSP70 and downregulation of BAX protein in rats pre-treated with the mushroom extract. Further studies are required to determine the bioactive components responsible for the mechanism of antiulcer activities of *H. erinaceus* extracts indicated in this study.

## Figures and Tables

**Table 1:** Effect of aqueous extract of *H. erinaceus* on different gastric parameters of ethanol-induced rats.

Animal groups	Treatment (5 mL/kg dose)	Ulcer area (mm^2^) Mean ± SEM	Ulcer inhibition (%)	Mucus production (g)
1	Non-treatment group	0	0	0.42 ± 0.06^∗#^
2	Ulcer control group	894.00 ± 36.69^#^	0	0.79 ± 0.03^#^
3	Omeprazole (20 mg/kg)	285.90 ± 19.64*	67.45	1.56 ± 0.07*
4	*H. erinaceus* (50 mg/kg)	727.20 ± 26.88^∗#^	18.14	0.79 ± 0.03^#^
5	*H. erinaceus* (100 mg/kg)	602.40 ± 29.43^∗#^	32.17	0.82 ± 0.04^#^
6	*H. erinaceus* (200 mg/kg)	453.60 ± 27.89^∗#^	48.60	0.93 ± 0.06^#^
7	*H. erinaceus* (400 mg/kg)	240.00 ± 17.31*	72.97	1.18 ± 0.04^∗#^

All values were expressed as mean ± standard error of mean. *Significant difference (*P* < 0.05) when compared with the ulcer control (group 2). ^#^Significant difference (*P* < 0.05) when compared with the omeprazole (group 3).
